# Robust Visual Ship Tracking with an Ensemble Framework via Multi-View Learning and Wavelet Filter

**DOI:** 10.3390/s20030932

**Published:** 2020-02-10

**Authors:** Xinqiang Chen, Huixing Chen, Huafeng Wu, Yanguo Huang, Yongsheng Yang, Wenhui Zhang, Pengwen Xiong

**Affiliations:** 1 Institute of Logistics Science and Engineering, Shanghai Maritime University, Shanghai 201306, China; chenxinqiang@stu.shmtu.edu.cn (X.C.); yangyssmu@163.com (Y.Y.); 2 Merchant Marine College, Shanghai Maritime University, Shanghai 201306, China; chenhuixing@stu.shmtu.edu.cn; 3 School of Electrical Engineering and Automation, Jiangxi University of Science and Technology, Ganzhou 341000, China; jxhuangyg@126.com; 4 School of Traffic and Transportation, Northeast Forestry University, Harbin 150040, China; 5 School of Information Engineering, Nanchang University, Nanchang 330031, China; steven.xpw@ncu.edu.cn

**Keywords:** visual ship tracking, multi-view learning, wavelet filter, data quality control, smart ship

## Abstract

Maritime surveillance videos provide crucial on-spot kinematic traffic information (traffic volume, ship speeds, headings, etc.) for varied traffic participants (maritime regulation departments, ship crew, ship owners, etc.) which greatly benefits automated maritime situational awareness and maritime safety improvement. Conventional models heavily rely on visual ship features for the purpose of tracking ships from maritime image sequences which may contain arbitrary tracking oscillations. To address this issue, we propose an ensemble ship tracking framework with a multi-view learning algorithm and wavelet filter model. First, the proposed model samples ship candidates with a particle filter following the sequential importance sampling rule. Second, we propose a multi-view learning algorithm to obtain raw ship tracking results in two steps: extracting a group of distinct ship contour relevant features (i.e., Laplacian of Gaussian, local binary pattern, Gabor filter, histogram of oriented gradient, and canny descriptors) and learning high-level intrinsic ship features by jointly exploiting underlying relationships shared by each type of ship contour features. Third, with the help of the wavelet filter, we performed a data quality control procedure to identify abnormal oscillations in the ship positions which were further corrected to generate the final ship tracking results. We demonstrate the proposed ship tracker’s performance on typical maritime traffic scenarios through four maritime surveillance videos.

## 1. Introduction

A smart ship is considered in the ship industry as having the advantages of less carbon emissions, lower risk for the ship crew at sea, higher traffic efficiency, larger cargo carriage capability, etc., and thus it has attracted much research attention in the maritime traffic community [1,2,3]. For the purpose of helping in smart ship maritime navigational environments, varied ship tracking techniques and data sources are employed to obtain informative static and kinematic ship data from varied maritime sources. For instance, ship tracking data from maritime surveillance videos provide straightforward spatial-temporal information (e.g., ship trajectory, ship speeds, ship moving directions) which greatly enriches the situational awareness capability of the smart ship and, thus, further improve maritime traffic safety. More specifically, by noticing potentially risky ship behaviors from the ship tracking results, the smart ship can inform the risk-involved ships to take early action (e.g., maneuver ship engines) to avoid potential maritime accidents.

Previous studies mainly employed automatic identification systems (AIS) to track ships sailing in inland waterways [4,5,6], and several techniques (e.g., synthetic aperture radar (SAR), long-range identification and tracking (LRIT)) have been integrated to further enhance ship tracking accuracy [7,8,9,10]. More specifically, maritime traffic participants can track ship positions with the LRIT technique over large time intervals (usually every six hours) when the ship travels far away from coastal areas. Ships sailing in inland waterway channels can be tracked at smaller time intervals with the help of AIS-relevant techniques. Indeed, a ship equipped with AIS facilities broadcasts the ship’s position to neighboring ships and stations every ten seconds; thus, the ship’s position in coastal areas is available at high resolution from the perspective of time scale (i.e., a ship’s positions in inland waters can be accessed at higher accuracy compared to those obtained by LRIT). The radar-relevant techniques can be integrated to improve ship position accuracy (e.g., a ship’s crew may intentionally deactivate their AIS facility when they are involved in illegal activities) [11,12,13,14]. With the help of human involvement, the abovementioned techniques can meet ship tracking demands in a traditional navigation period. More specifically, maritime traffic participants are required to transform the abovementioned ship data (which are considered second-hand maritime visual data) into human-understandable sources (which are considered first-hand maritime visual data) in traditional navigation period. However, the ship crew on board will be significantly reduced in the smart ship era, which imposes additional challenges for autonomous ship tracking tasks (i.e., efficiently obtaining first-hand visual tracking data with less human involvement). 

Computer vision-based methods have shown many favorable results in the object tracking community [15,16,17,18] which presents its potential in addressing the above challenge (i.e., obtaining first-hand visual data). More specifically, image-based ship tracking models have attempted to extract distinct features to identify ship positions from maritime image sequences. Prasad et al. [18] provided a holistic literature review on ship detection and tracking which can be described in consecutive steps: horizontal line detection, background subtraction, and foreground segmentation, and is considered the mainstream workflow for maritime object detection. Hu et al. [19] proposed a defogging framework for the purpose of removing fog interference in a single maritime image which may fail to obtain haze-removal maritime images in real-world applications. Leclerc et al. [15] employed a deep convolution neural network to learn distinct ship features from maritime images which can be used for tracking ships in maritime images. We did not focus on deep learning model performance for ship tracking tasks, although they have shown many successes in general purpose computer vision tasks [20,21]. The main reason is that many ship training samples (usually collected in a manual manner) are required to train deep learning models, and the on-board device may lack sufficient computational resource when implementing ship tracking tasks in real-world maritime applications. 

Park et al. [22] proposed a passive ship tracking model to accurately extract ship trajectories from maritime videos shot by onboard monocular camera. Wawrzyniak et al. [23] integrated the background subtraction and bounding box methods to track ships in maritime video streams which was demonstrated with typical ship tracking challenges. Kang et al. [24] proposed a self-selective correlation filtering method to track ships by tackling the challenges of ship imaging size variation and background interference. Zhang et al. [25] proposed a discrete cosine transformation-based ship detection framework to obtain ship trajectories from maritime images shot by non-stationary platform cameras. Similar studies can be found in References [26,27,28]. 

Previous models fulfilled the visual ship tracking task by extracting a group of distinct ship features (e.g., intensity, edge, contours, texture, color) from maritime video clips. Following the rule in the object tracking community, visual feature-based ship tracking models may suffer from the intrinsic weakness of a 20 pixel error [29]. More specifically, previous studies considering the ship tracking models obtained 100% accuracy when the average Euclidean distance between the tracked and ground truth (i.e., manual labeled) ship positions did not exceed 20 pixels. However, the 20 pixel error may indicate abnormal ship position oscillations which are supposed to be trivial tracking outliers. To address the issue (i.e., alleviate the trivial tracking oscillations), we propose an ensemble framework by integrating multi-view learning and data quality control procedures to robustly track ships from maritime images. The proposed framework includes three steps which are sampling varied ship training candidates with a particle filter (PF), obtaining raw ship tracking results with a multi-view learning model, and trivial position oscillation removal with the wavelet filter (WF). The remainder of this study is organized as follows. The proposed ensemble ship tracking framework based on multi-view learning and the WF model is introduced in detail in Section 2. The dataset and experimental results are described in Section 3. We briefly conclude our study in Section 4. 

## 2. Methodology

### 2.1. Framework Overview 

**Figure 1:**
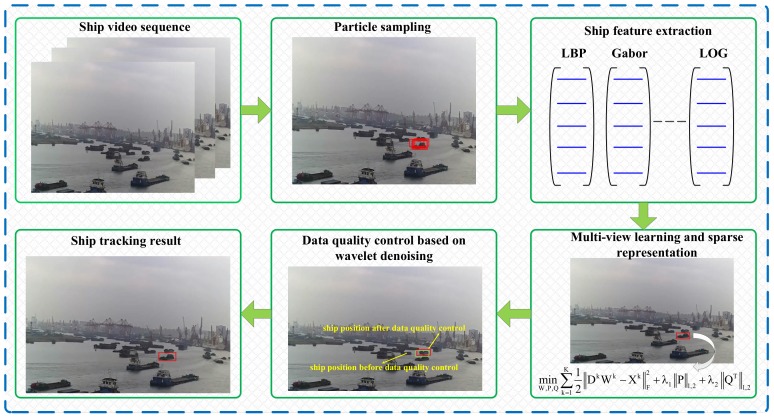
Schematic diagram of the proposed ship tracking framework. LOG = Laplacian of Gaussian. LBP = local binary pattern.

The flowchart of the proposed framework is shown in Figure 1. Ship training samples are crucial for the success of the ship tracking task which were transformed into the ship state prediction in maritime image sequences in our study. The PF algorithm randomly samples and re-samples the region of interest (ROI) in the input frames for the purpose of generating a group of particles (serving as inputs for training the tracking model) and has shown many successful broad ranges of state estimation applications in the object tracking community (including the ship state prediction task) [30]. In that manner, we employed the PF model to estimate potential ship moving states (i.e., ship training candidates) in the ROI from maritime images. Then, the multi-view learning model was proposed to learn the distinctive ship features from the outputs of the previous step and determine the raw ship tracking results (i.e., to-be-tracked ship position) in maritime images. Finally, we implemented a data quality control procedure to identify anomalous ship positions (i.e., abnormal position oscillations) and suppress the unexpected outliers with the WF algorithm. For the convenience of readability, our proposed ensemble ship tracking framework is abbreviated as MVLWD (i.e., multi-view learning and wavelet de-noising) in the following sections. 

### 2.2. Ship Training Candidates Sampling with PF Model 

The PF algorithm is considered a type of Monte Carlo method which generates ship candidates from maritime images with sequence importance sampling criterion. More specifically, the target ship of the ROI area is sampled by the PF model at a higher probability and vice versa. Note that the PF model generates varied ship training samples by estimating posterior probability distribution based on the initial target ship position (which is manually labeled in the first frame). For a given ship video clip, we employed $x_{t}$ to represent the ship position state at maritime frame t and the observed ship positions from frame 1 to frame t-1 are denoted as $y_{1:t-1}=\left\{y_{1},\ldots ,y_{t-1}\right\}$. The probability distribution of $x_{t}$ can be predicted with the previously observed ship positions $y_{1:t-1}$, which is formulated as follows:

(1) $$p(x_{t}|y_{1:t-1})=\int p(x_{t}|x_{t-1})p(x_{t-1}|y_{1:t-1}){\mathrm{dx}}_{t-1}$$

where the parameter $p(x_{t}|x_{t-1})$ is the ship state transition distribution likelihood, and the parameter t should be larger than 1 (i.e., we did not estimate the ship state transition in the first frame used in the PF model initialization procedure). 

Following the rule in previous studies [31,32], we can obtain the observed ship position $y_{t}$ at frame t, and thus the posterior probability distribution for the updated ship position state $x_{t}$ is shown as follows:

(2) $$p(x_{t}|y_{1:t})=\frac{p\left(y_{t}{|x}_{t}\right)p(x_{t}|y_{1:t-1})}{p(y_{t}|y_{1:t-1})}$$

where $p(y_{t}|x_{t})$ is the observation likelihood for a ship position state $x_{t}$, and $p(y_{t}|y_{1:t-1})$ is a constant. 

The PF model estimates the posterior probability $p(x_{t}|y_{1:t})$ from a group of particles ${\left\{x_{t}^{r}\right\}}_{r=1,\ldots ,n}$ (each particle is a ship candidate in the current ship frame), and each particle is assigned a weight $w_{t}^{r}$ (*n* is the particle number). The weight $w_{t}^{r}$ in the frame t is updated with observation likelihood $p(y_{t}|x_{t})$ according to the bootstrap filter strategy (see Equation (3)). Note that the observation likelihood for ship position state $x_{t}$ quantitatively measures the similarity between a particle (i.e., a ship candidate) in the frame t and the ship training template.

(3) $$w_{t}^{r}=w_{t-1}^{r}p(y_{t}|x_{t})$$

### 2.3. Ship Tracking with Multi-View Learning 

After obtaining the ship candidates in the previous step, the proposed MVLWD framework implements the multi-view learning model to track targets by exploiting high-level distinct ship features (e.g., shape, contour, texture) from the PF sampling results. More details are described in the following. 

#### 2.3.1. Ship Feature Extraction

Previous studies suggest that extracting holistic and distinct feature sets are crucial for the ship tracking task [26,33,34]. The main reason is that a ship’s visual appearance in maritime images may experience various imaging challenges such as illumination, deformation, and occlusion. In that manner, a single type of ship feature may fail to obtain favorable tracking results. Contour and texture-relevant features have shown favorable performance in many computer vision tasks (e.g., vehicle tracking, pedestrian trajectory extraction) [35,36]. Moreover, ship contours and textures are significantly different from those of water in maritime image sequences. Based on the abovementioned analysis, we employed texture- and edge-based feature descriptors to extract distinct ship appearance features in the ROI of each maritime image. The histogram of oriented gradient (HOG), local binary pattern. (LBP), and Gabor feature descriptors are commonly used to identify target contours for the purpose of helping varied trackers obtain high tracking accuracy which indeed have enjoyed huge success in many object tracking applications [10]. The Canny feature descriptor can effectively overcome the edge variation challenge (caused by wave imaging changes) [37], and the LoG descriptor is a popular blob-detector providing a complementary description of ship shape [38]. The MVLWD ship tracker extracts the above five ship edge features which are further learned by the multi-view learning model to obtain the compact and high-level ship features. For the purpose of methodology generalization, we set the symbol G to represent the number of features in our proposed MVLWD tracker. 

#### 2.3.2. Establishing Ship Tracking Model with Multi-View Learning 

Ship tracking model is required to robustly track ships under varied tracking challenges in typical maritime traffic scenarios. To that aim, several studies have been conducted to extract different shallow ship features from the ship training samples (the intrinsic correlations among ship feature sets may not be fully exploited) which are used to identify target ships in maritime images [39,40]. To bridge the gap, we employed the multi-view learning model to couple varied ship features into a high-level compact ship descriptor. Note that each view in the multi-view learning model is indeed a distinct feature. 

We employed *n* particles to sample the ship images (i.e., each image generates *n* ship training candidates), and each sample contained G different features (i.e., ship edge and texture features obtained in the previous step). Thus, we needed to solve n×G ship tracking tasks considering that the learning sparse representation of each feature of a particle is an individual task. For each feature, we employ g = 1,…, G, $X^{g}\in {\mathbb{R}}^{d_{g}\times n}$ to represent the ship feature matrix, where $d_{g}$ is the feature vector dimension for the corresponding gth feature (i.e., the gth ship feature). We denoted $D^{g}\in {\mathbb{R}}^{d_{g}\times N}$ as an over-complete ship dictionary, where each column of $D^{g}$ is the ship template of the gth feature, and *N* represents the number of ship templates. Following the rule in Reference [41], we can obtain the relationship between the latent representation matrix $W^{g}$ and the ship view matrix $X^{g}$. Given the ship feature matrices $X^{g}=\left\{X^{1},\ldots ,X^{G}\right\}$ for the *n* particles and the corresponding training sample responses $Y^{g}=\left\{Y^{1},\ldots ,Y^{G}\right\}$, we can establish a linear function between the ship feature matrices and response with $Y^{g}\approx X^{g}W^{g}$ (g = {1, 2, …, G}). In that manner, we obtained the latent representation matrices $W^{g}=\left\{W^{1},\ldots ,W^{G}\right\}$ (the $W^{g}$ is also known as decomposition matrix).

The $W^{g}$ explores the independence for the ship particle by capturing the intrinsic statistical characteristics among different features. Note that each $W^{g}$ can be decomposed into two collaborative components $P^{g}$ and $Q^{g}$, which is formulated as $W^{g}=P^{g}+Q^{g}$. The $P^{g}$ is considered as the weight matrix on the row sparse constraint, and the $Q^{g}$ is the counterpart of the column matrix. More specifically, the *z*th column of $W^{g}$ is non-zero when the *z*th column for $Q^{g}$ is non-zero. In that manner, we considered the *z*th ship particle as an outlier task. At the same time, the zero columns of $Q^{g}$ indicated that the non-zero rows of $P^{g}$ shared the same ship features. After decomposing the representation matrices for all of the ship features, we padded the weight matrices $P^{g}$ and $Q^{g}$ (g = 1, …, G) in a horizontal manner. In that manner, we obtained global weight matrices P and Q on the row and column directions, respectively. We introduced the group least absolute shrinkage and selection (LASSO) penalty method (denoted as $\ell_{1,2}$) to minimize the feature difference between different tasks and the corresponding features [42]. More specifically, we obtained the joint distinct ship features and suppressed the trivial features on the row matrix (i.e., the P matrix) with the $\ell_{1,2}$ criterion, and the corresponding outputs were the sparse representation matrix for the P. Note that the rule was applicable to the column matrix (i.e., Q matrix). The multi-view learning-based representations for the sampled ship particles can be obtained by solving Equation (4):

(4) $$\underset{W,P,Q}{\min}\overset{G}{\underset{g=1}{\sum }}f_{L}\left(D^{g}W^{g}-X^{g}\right)+\lambda_{1}\|P{\|}_{1,2}+\lambda_{2}\|Q^{T}{\|}_{1,2}$$

where $f_{L}\left(D^{g}W^{g}-X^{g}\right)$ is the cost function for the reconstruction errors during multi-view learning procedure. The parameters $\lambda_{1}$ and $\lambda_{2}$ control the sparsity of the matrices *P* and *Q*, respectively. The operator $\|•{\|}_{1,2}$ is equal to ∑i∑j·i,j212, where ${\left(\cdot \right)}_{i,j}$ is the matrix element at the *i*th row and *j*th column. 

Previous studies suggest that the cost function in Equation (4) plays a crucial role for the performance of the multi-view learning-based tracker [43]. The Frobenius norm has shown its advantages in different tracking applications, which is employed as the cost function in our study too. Thus, we can re-formulate Equation (4) by substituting the cost function with the Frobenius norm which is shown as follows:

(5) $$\underset{W,P,Q}{\min}\overset{G}{\underset{g=1}{\sum }}\frac{1}{2}\|D^{g}W^{g}-X^{g}{\|}_{F}^{2}+\lambda_{1}\|P{\|}_{1,2}+\lambda_{2}\|Q^{T}{\|}_{1,2}$$

where $\|•{\|}_{F}^{2}$ is the Frobenius norm. 

#### 2.3.3. Solving the Ship Tracking Model 

The multi-view learning-based ship tracking performance (i.e., the tracking accuracy) heavily relies on the solution for Equation (5). For the purpose of readability convenience, we split the Equation (5) into two parts which are shown as Equations (6) and (7). More specifically, we can obtain the optimal solution for Equation (5) by combing the solutions of Equations (6) and (7) in a linear superposition manner. Previous studies suggested that the accelerated proximal gradient (APG) method can successfully tackle the Frobenius-norm relevant optimization challenges [44] and thus is employed in our study too. The residual of ship tracking results obtained by the APG model at the *v*th iteration is $Ο\left(\frac{1}{v^{2}}\right)$, and thus we can obtain a smaller tracking error by implementing the APG model with more iterations.

(6) $$\varphi \left(P,Q\right)=\overset{G}{\underset{g=1}{\sum }}\frac{1}{2}\|D^{g}W^{g}-X^{g}{\|}_{F}^{2}$$

(7) $$s\left(P,Q\right)=\lambda_{1}\|P{\|}_{1,2}+\lambda_{2}\|Q^{T}{\|}_{1,2}$$

The APG method solves Equations (6) and (7) with the steps of composite gradient mapping and aggregation which are detailed as follows: 

Step 1: Composite gradient mapping. Motivated by previous studies [32,45], we introduced the composite gradient mapping model to address the ship tracking problem in Equation (5). More specifically, we reformulated the ship tracking problem with Equations (5)–(7) in the form of Equation (8) as follows:

(8) $$\Gamma \left(P,Q;A,B\right)=\varphi \left(A,B\right)+\langle \nabla_{A}\varphi \left(A,B\right),P-A\rangle +\langle \nabla_{B}\varphi \left(A,B\right),Q-B\rangle \;+\frac{\gamma }{2}\|P-A\|{\;}_{F}^{2}+\frac{\gamma }{2}\|Q-B{\|}_{F}^{2}+s\left(P,Q\right)$$

where $\varphi \left(A,B\right)$ is the first-order Taylor expansion of $\varphi \left(P,Q\right)$ at point $\left(A,B\right)$. The $s\left(P,Q\right)$ is the regularization term which is presented with the Euclidean distance between points $\left(P,Q\right)$ and $\left(A,B\right)$. The parameter γ represents the penalty coefficient for each iteration. In particular, $\nabla_{A}\varphi \left(A,B\right)$ and $\nabla_{B}\varphi \left(A,B\right)$ represent the partial derivatives of $\varphi \left(A,B\right)$ with respect to A and B. The $\langle \nabla_{A}\varphi \left(A,B\right),P-A\rangle$ is the inner product between $\nabla_{A}\varphi \left(A,B\right)$ and P−A, and $\langle \nabla_{B}\varphi \left(A,B\right),Q-B\rangle$ represents the inner product of $\nabla_{B}\varphi \left(A,B\right)$ and Q−B. 

Step 2: Aggregation. At the *v*th APG iteration procedure, $\left(A^{v+1},B^{v+1}\right)$ can be calculated by linearly combining $\left(P^{v},Q^{v}\right)$ and $\left(P^{v-1},Q^{v-1}\right)$. Thus, we can obtain the updated $A^{v+1}$ and $B^{v+1}$ by Equations (9) and (10), respectively. The parameter $\eta_{v}$ is identified with Equation (11). Note that each element in the matrices $P^{0}$, $Q^{0}$, $A^{1}$, and $B^{1}$ are set to zero for the purpose of initialization.

(9) $$A^{v+1}=P^{v}+\eta_{v}\left(\frac{1-\eta_{v-1}}{\eta_{v-1}}\right)\left(P^{v}-P^{v-1}\right)$$

(10) $$B^{v+1}=Q^{v}+\eta_{v}\left(\frac{1-\eta_{v-1}}{\eta_{v-1}}\right)\left(Q^{v}-Q^{v-1}\right)$$

(11) $$\eta_{v}=\left\{\begin{array}{cc} \frac{2}{v+3}, & v\geq 1\\ 1, & v=0 \end{array}\right.$$

Given the set $\left(A^{v},B^{v}\right)$, the solution of the *v*th iteration of the APG procedure is to find the minimal solution for Equation (12) which can be decomposed as finding the minimization values for the $P^{v}$ and $Q^{v}$ (see the Equations (13) and (14), respectively). Following the rules in Reference [46], we obtained the solutions for each row of P and each column of Q in Equations (13) and (14) with a closed form solution through Equations (15) and (16), respectively. More specifically, the ship tracking result was obtained when the minimization problems in Equations (13) and (14) were solved.

(12) $$\left(P^{v},Q^{v}\right)=\underset{P,Q}{\mathrm{arg min}}\Gamma \left(P,Q;A^{v},B^{v}\right)$$

(13) $$P^{v}=\underset{P}{\mathrm{arg min}}\frac{1}{2}\|P-R^{v}{\|}_{F}^{2}+\frac{\lambda_{1}}{\gamma }\|P{\|}_{1,2}$$

(14) $$Q^{v}=\underset{Q}{\mathrm{arg min}}\frac{1}{2}\|Q-S^{v}{\|}_{F}^{2}+\frac{\lambda_{2}}{\gamma }\|Q^{T}{\|}_{1,2}$$

(15) $$P_{i,\cdot }^{v}=\max(0,1-\frac{\lambda_{1}}{\gamma \|R_{i,\cdot }^{v}\|})R_{i,\cdot }^{v}$$

(16) $$Q_{\cdot ,j}^{v}=\max(0,1-\frac{\lambda_{2}}{\gamma \|S_{\cdot ,j}^{v}\|})S_{\cdot ,j}^{v}$$

where $P_{i,\cdot }^{v}$ represents the *i*th row of $P^{v}$ and $Q_{\cdot ,j}^{v}$ represents the *j*th column of $Q^{v}$. The operator ‖•‖ is the Euclidean distance.

### 2.4. Ship Position Denoising with WF Model 

The outputs from the previous step are the raw ship tracking positions which may contain small tracking errors such as irregular ship position oscillations. The main reason for causing such anomalous tracking results can be ascribed to the low video quality and background imaging interference (e.g., waves, buoys). The WF model is thus applied to rectify the potential outliers which is detailed described as follows. Note that ship tracking position in each frame was represented as $C_{x}$, $C_{y}$, $C_{w}$, $C_{h}$ where $C_{x}$ and $C_{y}$ are the *x* and *y* coordinates for the top-left point of the bounding box (i.e., the tracked ship). The $C_{w}$ and $C_{h}$ are the width and height for the bounding box, respectively. We describe the WF denoising procedure on the $C_{x}$ data series for the sake of simplicity (the rule is applicable to the $C_{y}$, $C_{w}$, and $C_{h}$ data series). We denoted the raw *x* coordinates by the previous step as S($C_{x}$), which was further processed by a J-scale decomposition of the WF model. The S($C_{x}$) can be decomposed into approximate coefficient $A_{c}$ and detail coefficients $D_{c}$ $\left(c=1,\ldots ,J\right)$, which is shown as follows:

(17) $$S\left(C_{x}\right)=A_{c}+\overset{J}{\underset{c=1}{\sum }}D_{c}$$

The detail coefficients $D_{c}$ are the details in the raw $C_{x}$ data series which contains both of the regular and irregular oscillations. We employed the soft threshold function to obtain noise-free detail coefficients ${\hat{D}}_{c}$ at varied scales (see Equations (18) and (19)) [47]. In that manner, we reconstructed the noise-suppressed *x* coordinates $\hat{S}\left(C_{x}\right)$ with Equation (20).

(18) $${\hat{D}}_{c}=\left\{\begin{array}{cc} \mathrm{sign}\left(\left(D_{c}\right)\right)\left(\left|D_{c}\right|-t_{g}\right), & \left|D_{c}\right|\geq t_{g}\\ 0, & \left|D_{c}\right|<t_{g} \end{array}\right.$$

(19) $$t_{g}=\sigma \sqrt{2\log\left(g\right)}$$

(20) $$\hat{S}\left(C_{x}\right)=A_{c}+\overset{J}{\underset{c=1}{\sum }}{\hat{D}}_{c}$$

where *g* is the sample length for the $C_{x}$ data series. The $t_{g}$ is the threshold for determining the anomaly detail coefficient and σ represents the standard deviation of the noise in the ${\;C}_{x}$ series.

## 3. Experiments

### 3.1. Data

**Figure 2:**
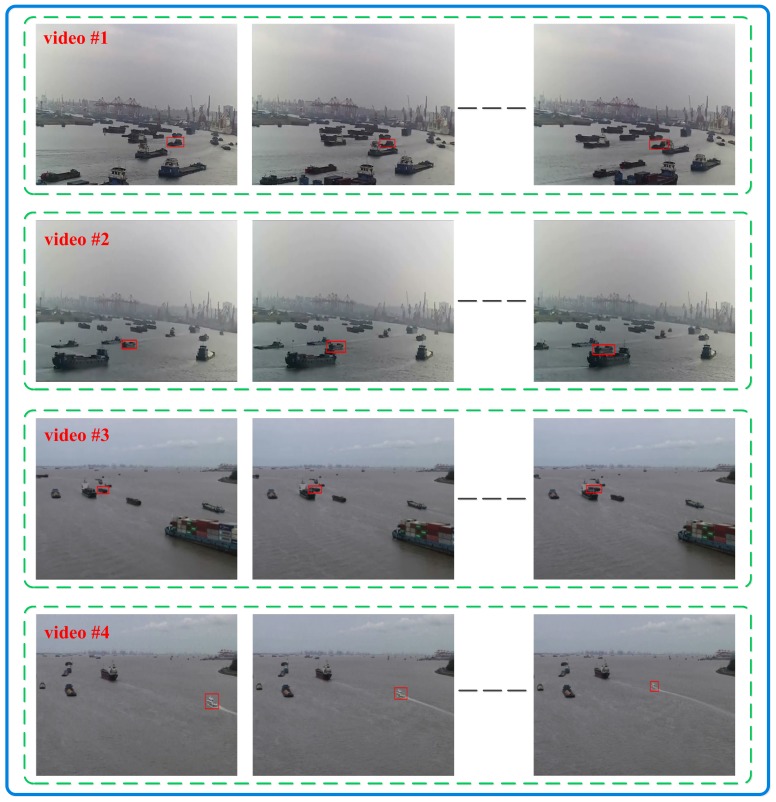
Ship frame samples for the collected video clips.

We performed the ship tracker on different maritime videos to verify the effectiveness of our proposed MVLWD model. More specifically, four video clips involved with typical maritime traffic scenarios were shot at Shanghai Port (denoted as video #1, #2, #3, and #4), which were used as the benchmark data for evaluating the proposed ship tracking model performance. The ship tracking challenge in each video was different from each other and is described in detail as follows. Videos #1 and #2 involved ship overlapping challenges in the two maritime video clips. More specifically, the visual appearance of the target ship was similar to that of the obstacle in video #1, and the overlapping-involved ships in video #2 showed different appearances. The main tracking challenge in video #3 is that the to-be-tracked ship was small in imaging size, and the target ship in video #4 was interfered with by the wave clutters in video #4. We are willing to share our collected maritime video clips with interested readers (by request) considering that public computer vision datasets (e.g., Visual Object Tracking (VOT) [48]) do not contain typical maritime traffic scenarios. The typical frames for each video clip are shown in Figure 2.

### 3.2. Experimental Platform and Evaluation Criteria

For the purpose of tracking performance validation, the proposed MVLWD ship tracker and another two popular ship trackers were implemented to track ships in the above four video sequences. The two ship tracking models were labeled as the Meanshift and ship tracker based on multi-view learning and sparse representation (abbreviated as STMS) [10,49]. The three trackers were implemented on Windows 7 OS, and the CPU was an Inter Core i5-4210M at 2.6 GHz and the RAM was 8 GB. The simulation platform was MATLAB (R2016 version). A previous study suggested that the precision and success rate can be used for evaluating a ship tracker’s performance [29]. The success rate relevant metrics can be easily identified in a manual manner and thus were not used in our study. In that manner, we employed precision relevant metrics to quantify the performance of the ship tracking model.

Note that the ground truth ship positions in the first frame of each video were employed to initialize the three trackers. To the aim of quantitatively evaluating the ship tracker’s performance, we employed the Euclidean distance to measure the distance between the ground truth and the tracked ship positions. More specifically, the ship (both the ground truth and tracked) are presented by a bounding box in ship frames, and the Euclidean distance between the center points of the ground truth and tracked ship position was calculated to evaluate the ship tracker’s performance. 

We used $G_{m}\left(x,y\right)$ to represent the ground truth ship position (i.e., the center point of the ground truth bounding box) at maritime frame m, and $T_{m}\left(x,y\right)$ was the counterpart (i.e., the tracked ship position). The Euclidean distance between the two points in frame m was calculated by Equation (21). Three typical statistical indicators were employed to further demonstrate the tracking model’s performance: mean distance (MD), root mean square error (RMSE), and mean absolute error (MAE) (see Equations (22)–(24)). A smaller value of MD indicates that the tracked ship position series was closer to the ground truth data (i.e., the ship tracking model obtained better accuracy) and vice versa. The rule is applicable to the RMSE and MAE statistics.

(21) $$E_{m}\left(G,T\right)=\sqrt{{\left(G_{m}\left(x\right)-T_{m}\left(x\right)\right)}^{2}+{\left(G_{m}\left(y\right)-T_{m}\left(y\right)\right)}^{2}}$$

(22) $$MD=\frac{1}{M}\sum_{m=1}^{M}E_{m}\left(G,T\right)$$

(23) $$RMSE=\sqrt{\frac{1}{M}\overset{M}{\underset{m=1}{\sum }}{\left(E_{m}\left(G,T\right)-MD\right)}^{2}}$$

(24) $$MAE=\frac{1}{M}\overset{M}{\underset{m=1}{\sum }}\left|E_{m}\left(G,T\right)-MD\right|$$

where *M* is the ship position sample length. The $\;G_{m}\left(x\right)$ represents ground truth ship position on the *x*-axis of frame m, and $G_{m}\left(y\right)$ is the counterpart on the *y*-axis. The $T_{m}\left(x\right)$ and $T_{m}\left(y\right)$ represent the *x* and *y* coordinate of the tracked ship position of frame m. 

### 3.3. Results

#### 3.3.1. Ship Tracking Results for Video #1

We present the ship tracking results with the three trackers in detail for video #1, and then verify the model’s performance on video #2, #3, and #4. More specifically, we demonstrated our proposed ship tracking model’s (i.e., MVLWD model) performance with different wavelet bases, which were further compared against the other two ship tracking models. The harr basis, db basis, sym basis, coif basis, and bior basis in the WF model demonstrated the efficacy on the traffic data smooth applications [50] which showed the potential of suppressing ship position outliers in our study. In that manner, our proposed model performances were presented in detail with the WF model implemented with the above wavelet basis. The MVLWD model implemented with the harr wavelet was labeled as MVLWD (harr), and this rule is applicable to the MVLWD (db), MVLWD (sym), MVLWD (coif), and MVLWD (bior). 

**Figure 3:**
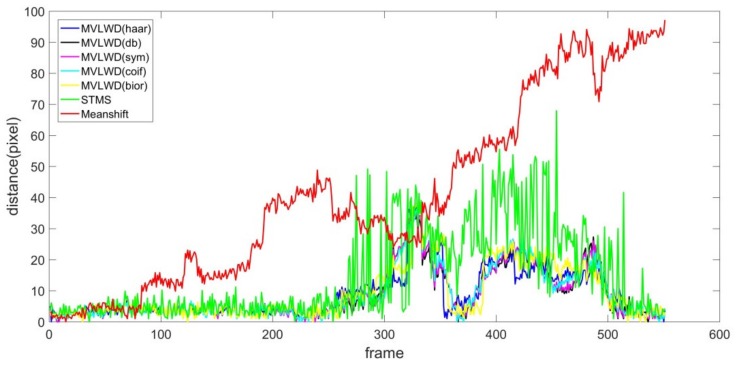
Euclidean distance distribution for the three trackers on video #1.

As shown by the red curve in Figure 3, we found that the tracking error of the Meanshift tracker was significantly larger than the other tracking models (i.e., the distance by the Meanshift tracker is obviously larger than the other two trackers). After carefully checking the Meanshift tracking results (by plotting the Meanshift tracking bounding boxes on the image sequences), we found that the Meanshift tracker wrongly tracked the target ship when it was partially occluded by the ship sailing in a neighboring waterway channel. The STMS Euclidean distance distribution curve (see the green line in Figure 3) fluctuated at smaller magnitude than that of the Meanshift tracker (though the STMS tracking accuracy at several frames were worse than those of Meanshift model). More specifically, the STMS tracker successfully tracked the target ship when the ship was partially occluded by the obstacles from frame #80 and on. It is noted that the STMS’s tracking performance, from approximately frame #250 to #520, was not satisfactory, as the target ship was completely sheltered by the neighboring ship. 

The proposed ship tracker’s performance (different wavelets are labeled with a different color) showed better accuracy as the corresponding Euclidean distances were lower than the counterparts of Meanshift and STMS trackers. From frame #1 to #250 (the target ship was not (or slightly) occluded by the neighboring ship), the proposed MVLWD tracker with different wavelets showed similar tracking accuracy to that of the STMS tracker. Our proposed ship tracker showed its advantages when the ship was severely occluded by the obstacle (see the Euclidean distance distributions from frame #250 to #520). The main reason is that the proposed ship tracker employed a data quality control procedure to validate ship moving displacements with the previous ship positions; thus, the abnormal oscillation data were successfully corrected. 

Table 1 showed that the MVLWD tracker (at different wavelet bases) obtained smaller tracking errors compared to the Meanshift and STMS trackers. More specifically, the MD obtained by the Meanshift tracker was 41.01 pixels which is approximately three-fold higher than that of the STMS tracker. The MD obtained by the proposed MVLWD tracker ranged from 8.46 pixels (obtained by the MVLWD with the db wavelet basis) to 9.15 pixels (obtained by the MVLWD (bior)) which demonstrates the efficacy of the proposed ship tracker. The RMSE and MAE statistics distributions for the three trackers show similar results to that of the MD. The RMSE and MAE indicators for the Meanshift tracker were 29.41 and 24.70 pixels, respectively, which are both higher than their counterparts for the STMS and MVLWD trackers. More specifically, the STMS obtained a, RMSE and MAE (14.16 and 11.93 pixels, respectively) that were both half of those of the Meanshift tracker, and the MVLWD counterparts were both half of that of its STMS counterparts. From the perspective of the RMSE and MAE, the MVLWD (haar) obtained the optimal tracking accuracy (i.e., the RMSE and MAE were 7.73 and 6.24 pixels, respectively). In summary, the proposed MVLWD tracker obtained better tracking accuracy on the ship occlusion challenge (with similar visual appearances shared by the target and obstacle ships) in video #1. Note that the MVLWD tracker with haar wavelet analysis was the default setting in our study without further specifications.

**Figure 4:**
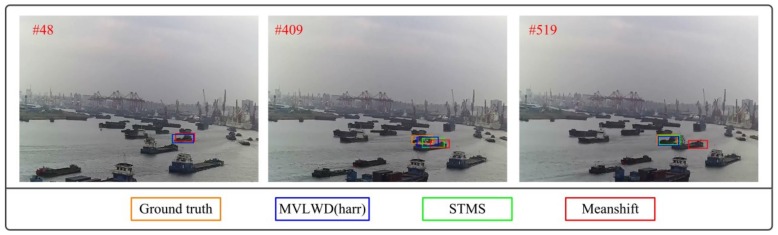
Ship tracking results for the three trackers on typical frames in video #1.

Typical tracking results on video #1 are shown in Figure 4 to further visualize the different ship trackers’ performance. It is noted that the three trackers showed favorable tracking accuracy at the beginning when the target ship was not occluded (see the ship tracking results at frame #48 in Figure 4). But, the two trackers (i.e., Meanshift and STMS) failed to accurately track the target ship in the latter frames (i.e., frames #409 and #519), as the tracked ship bounding boxes were not well matched with the ground truth. The main reason is that the two models extracted visual features (serving as ship descriptor) from the ship image sequences which are sensitive to the ship occlusion interference (i.e., the features may be wrongly extracted from neighboring ships). The proposed MVLWD tracker alleviated the ship occlusion interference by introducing spatial-temporal information in maritime images. More specifically, the WF model in the MVLWD decomposed the tracked ship’s positions into different details, where the noise details were suppressed from ship positions considering the spatial-temporal constraints. 

#### 3.3.2. Ship Tracking Results on Video #2

**Figure 5:**
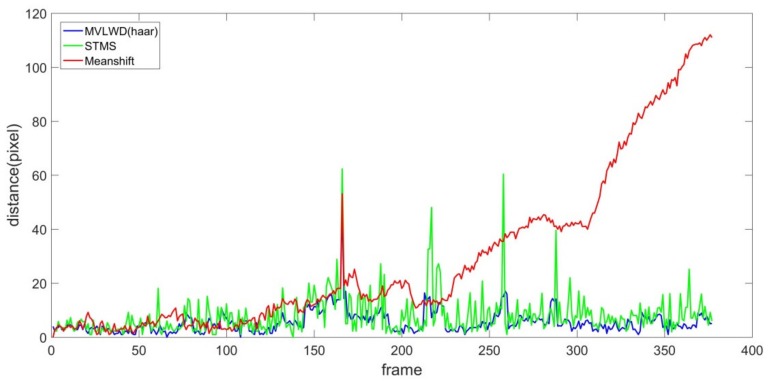
Euclidean distance distribution for the three trackers for video #2.

Video #2 involved a ship occlusion challenge, where the target ship was occluded by a neighboring ship with different visual appearances. The Euclidean distance distribution curve obtained by the Meanshift tracker showed a similar variation tendency as that of video #1 (see Figure 5). More specifically, the Meanshift tracker permanently lost the target ship due to the fact of a wrongly extracted shallow edge feature from the obstacle ship in the ship occlusion images. The obstacle ship was wrongly learned as template in the ship occlusion images which cannot be rectified in the latter tracking image sequences (i.e., the ship’s visual appearance can be fully observed without occlusions). The Euclidean distance distributions for the STMS and MVLWD trackers showed more stable performances in comparison to the Meanshift model. The main reason is that the two models extracted different ship features and thus ship occlusion with different visual features in video #2 can be partially suppressed during the ship tracking procedure. Besides, we found that the MVLWD tracker-obtained Euclidean distance distribution curve varied smoother than that of the STMS curve, and this indicates that the abnormal ship tracking oscillations were successfully suppressed. 

**Figure 6:**
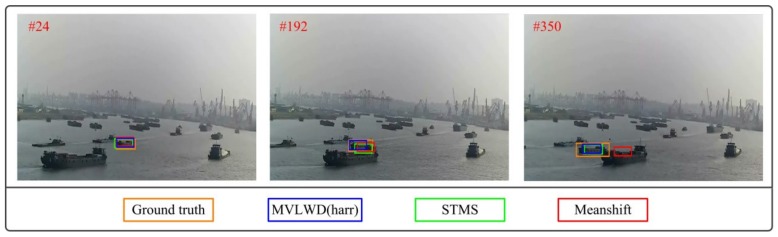
Ship tracking results for the three trackers for typical frames in video #2.

The statistical performances for the three trackers are shown in Table 2 which clearly demonstrates that the MVLWD tracker obtained better tracking accuracy than the other two trackers. More specifically, the three statistical indicators (i.e., MD, RMSE, and MAE) for the MVLWD tracker were 5.44, 4.16, and 2.80 pixels. The statistical values for the STMS model were at least 30% higher than the counterparts of the MVLWD tracker, and the Meanshift tracking accuracy were significantly lower than those of the two trackers. The typical ship tracking results on video #2 were shown in Figure 6 which were different from those shown in video #1. More specifically, the three trackers were not severely degraded by the ship occlusion challenge when the obstacle ship showed different visual appearances in comparison to the target ship. The Meanshift tracker slightly lost the target ship (i.e., the Meanshift bounding boxes in the frames were not far away from the ground truth) when the ship occlusion happened. Both the STMS tracker and MVLWD tracker successfully tracked the target ship in the ship occlusion frames (see the tracking results for frames #192 and #350), while the proposed ship tracker obtained closer ship tracking results. The ship tracking results in video #1 and #2 showed that our proposed MVLWD tracker can suppress ship occlusion interference and obtain better tracking results in comparison to the other two trackers.

#### 3.3.3. Ship Tracking Results for Videos #3 and #4

**Figure 7:**
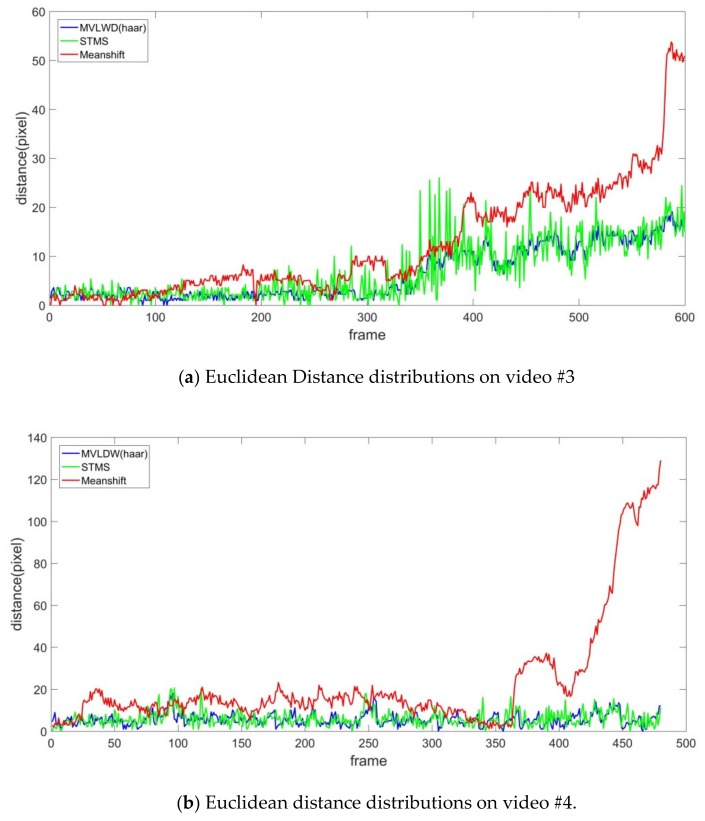
Euclidean distance distribution for the three trackers on video #3 and video #4.

The proposed MVLWD and the two ship trackers were also implemented to tackle the challenge of small ship tracking and sea clutter interference (i.e., videos #3 and #4, respectively). The Euclidean distance distribution shown in Figure 7 indicates that the MVLWD tracker obtained better tracking accuracy than the Meanshift and STMS models on the two typical ship tracking challenges. The Meanshift tracker identified background and clutter pixels near the target ship as the ship which were thus wrongly tracked in the maritime images, leading to the performance loss for the Meanshift tracker (see the red curves in Figure 7a,b). The STMS model was not obviously interfered by the tracking challenges in video #3 and #4 which can be observed in the green curve shown in Figure 7a,b, respectively. By contrast, our proposed ship tracker successfully determined intrinsic ship features from the maritime video clips, and the WF model successfully removed the trivial position oscillation outliers. 

We further analyzed ship trackers’ performances with the three statistical indicators which are shown in Table 3 and Table 4. It was found that the Meanshift model performance on video #3 was better than those on video #4 which is consistent with ship tracking results for the previous video clips. Based on that, we can conclude that the Meanshift tracking model performance easily suffered from the success/failure of ship visual feature extraction. It is noted that the MVLWD tracker obtained the minimum number of tracking errors for the two videos. More specifically, the MD, RMSE, and MAE obtained by the MVLWD tracker were 6.34 pixels, 5.22 pixels, 4.78 pixels on video #3 and 5.57 pixels, 2.93 pixels, 2.28 pixels on video #4 (which can be found in Table 3 and Table 4, respectively). According to the above analysis, our proposed ship tracker can successfully track ships at different ship tracking challenges in the four typical maritime video clips in the manner of obtaining robust ship features (and further discarding the trivial features from imaging interferences), eliminating the ship tracking outliers by suppressing the abnormal ship oscillations. 

## 4. Conclusions

Robust ship tracking plays a crucial role in visual perception tasks in the smart ship era, which can encounter varied challenges (ship occlusion, wave clutter interference, abnormal ship position variation, etc.). To address this issue, we proposed an ensemble ship tracking framework based on the multi-view learning algorithm and WF model. Firstly, the proposed ensemble ship tracking framework was used to generate ship candidates by sampling the maritime images with PF. Secondly, the multi-view learning model was proposed to track ships consisting of steps of feature extraction and ship tracking model establishment and solution determination. Thirdly, the data quality control procedure was implemented with the WF model to remove the abnormal tracking oscillations. We tested our proposed ship tracking model’s performance on the four typical scenarios (i.e., four typical ship tracking challenges), and the average MD, RMSE, and MAE for the proposed ship tracker were 6.47 pixels, 5.01 pixels, 4.03 pixels which were at least 20% lower than the counterparts for the Meanshift and STMS trackers. 

Though the proposed framework obtained favorable ship tracking performances for the typical maritime scenarios, we can further expand our research in the following aspects. First, the weather conditions in the collected ship video clips were relatively good (e.g., high visibility, windless). Testing the model tracking performance on the videos shot at extreme weather conditions can provide more holistic evaluation results. Second, our model worked well under a single ship tracking challenge in the four traffic scenarios and evaluation of the model’s performance with multiple ship tracking challenges is in need. Third, testing the ship’s tracking performance on a single maritime video involving several challenges can be an interesting exploration in the future. Fourth, exploiting the ship tracker’s performance with a non-perfect initialization challenge (instead of initializing with ground truths) can be another interesting expansion. Last but not least, we can further implement additional general-purpose tracking models (e.g., deep learning-based models, kernelized correlation filter for the purpose of tracker performance comparison. 

## Figures and Tables

**Table 1:** Statistical performances for the three trackers on video #1.

	MD (Pixels)	RMSE (Pixels)	MAE (Pixels)
Meanshift	41.01	29.41	24.70
STMS	14.03	14.16	11.93
MVLWD (haar)	8.54	7.73	6.24
MVLWD (db)	8.46	7.94	6.55
MVLWD (sym)	8.57	8.08	6.64
MVLWD (coif)	8.74	8.11	6.67
MVLWD (bior)	9.15	8.41	7.12

**Table 2:** Statistical performances of the three trackers for video #2.

	MD (Pixels)	RMSE (Pixels)	MAE (Pixels)
Meanshift	29.15	30.21	24.15
STMS	7.87	7.29	4.78
MVLWD (haar)	5.44	4.16	2.80

**Table 3:** Statistical performances for the three trackers on video #3.

	MD (Pixels)	RMSE (Pixels)	MAE (Pixels)
Meanshift	12.33	11.45	9.40
STMS	6.83	5.88	5.09
MVLWD (haar)	6.34	5.22	4.78

**Table 4:** Statistical performances for the three trackers on video #4.

	MD (Pixels)	RMSE (Pixels)	MAE (Pixels)
Meanshift	23.28	26.92	17.17
STMS	5.99	3.60	2.79
MVLWD (haar)	5.57	2.93	2.28
